# Citizen and stakeholder led priority setting for long-term care research: identifying research priorities within the Translating Research in Elder Care (TREC) Program

**DOI:** 10.1186/s40900-020-00199-1

**Published:** 2020-05-15

**Authors:** Stephanie A. Chamberlain, Carole A. Estabrooks, Janice M. Keefe, Matthias Hoben, Charlotte Berendonk, Kyle Corbett, Andrea Gruneir

**Affiliations:** 1 Department of Family Medicine, Faculty of Medicine & Dentistry, University of Alberta, Edmonton, Alberta T6G 2T4 Canada; 2 Faculty of Nursing, University of Alberta, Edmonton, Alberta T6G 1C9 Canada; 3 Department of Family Studies and Gerontology, Mount Saint Vincent University, Halifax, Nova Scotia B3M 2J6 Canada; 4 Translating Research in Elder Care (TREC) program, University of Alberta, Edmonton, Alberta T6G 1C9 Canada

**Keywords:** Citizen engagement, Stakeholder engagement, Long-term care, Priority setting, Health services research

## Abstract

**Background:**

The Translating Research in Elder Care (TREC) program is a longitudinal partnered program of research in Western Canada that aims to improve the quality of care and quality of life for residents and quality of worklife for staff in long-term care settings. This program of research includes researchers, citizens (persons living with dementia and caregivers of persons living in long-term care), and stakeholders (representatives from provincial and regional health authorities, owner-operators of long-term care homes). The aim of this paper is to describe how we used priority setting methods with citizens and stakeholders to identify ten priorities for research using the TREC data.

**Methods:**

We adapted the James Lind Alliance Priority Setting Partnership method to ensure our citizens and stakeholders could identify priorities within the existing TREC data. We administered an online survey to our citizen and stakeholder partners. An in-person priority setting workshop was held in March 2019 in Alberta, Canada to establish consensus on ten research priorities. The in-person workshop used a nominal group technique and involved two rounds of small group prioritization and one final full group ranking.

**Results:**

We received 72 online survey respondents and 19 persons (citizens, stakeholders) attended the in-person priority setting workshop. The workshop resulted in an unranked list of their ten research priorities for the TREC program. These priorities encompassed a range of non-clinical topics, including: influence of staffing (ratios, type of care provider) on residents and staff work life, influence of the work environment on resident outcomes, and the impact of quality improvement activities on residents and staff.

**Conclusions:**

This modified priority setting approach provided citizens and stakeholders with an opportunity to identify their own research priorities within the TREC program, without the external pressures of researchers. These priorities will inform the secondary analyses of the TREC data and the development of new projects. This modified priority setting may be a useful approach for research teams trying to engage their non-academic partners and to identify areas for future research.

## Plain English summary

Priority setting exercises have become a popular strategy for engaging patients, the public, and care providers, in identifying top priorities for research. Recently, a number of priority setting exercises have been done that focus on age-related conditions. However, these priority settings did not focus on any specific care setting. Research in long-term care homes (also known as nursing homes, personal care homes) is critical because the quality of care in these settings is often poor. The Translating Research in Elder Care (TREC) Program is a partnered program of research in three Western Canadian provinces (British Columbia, Alberta, Manitoba) that aims to improve the quality of care, quality of life, and quality of work life in long-term care homes. The program includes academic researchers, citizens (persons with a dementia, persons caring for someone in long-term care), and stakeholders (provincial ministries of health, regional health authorities, operators of long-term care homes). This study describes how we used priority setting methods to engage our citizen and stakeholder team members to identify research priorities within the TREC program.

We invited our citizens and stakeholder team members to complete an online survey and attend an in-person workshop. At the workshop, attendees generated a list of ten priorities for long-term care research based on existing TREC data. We received positive feedback from the citizens and stakeholders who participated in the in-person workshop. We believe that this priority setting exercise is a useful process for research teams to engage their non-academic partners to direct future projects and to advance research in long-term care.

## Background

Citizen engagement is increasingly recognized as a critical element in health research. As experts in their own lives [1], citizen collaboration is thought to enhance research relevance, improve the clarity of research products [2], and facilitate the dissemination of evidence [3]. Priority setting exercises have become one popular strategy for engaging research end users, typically citizens and care providers, in identifying and shaping relevant research questions. They have been increasingly used for identifying research questions related to older adults. Recent examples include priority settings co-led by the James Lind Alliance for the Alzheimer Society of Canada [4], the Canadian Frailty Network [5], and the Dementia Priority Setting Partnership in the United Kingdom [6]. While these efforts have helped to highlight the research needs of specific aging-related health issues, research in long-term care (LTC) settings (also known as nursing homes or personal care homes) has not been well represented.

LTC homes are the designated site of care for adults who cannot live safely in the community and require 24-h access to nursing and supportive care [7]. LTC residents are a highly vulnerable segment of the population. More than half are over age 80, upwards of 70% have a diagnosis of Alzheimer’s disease or other dementia, and multiple comorbid medical conditions are common [8–10]. Identifying priorities for research in LTC homes is critical because of the poor quality of care in these settings that has been widely reported both within Canada and abroad [11–13]. Poor quality of care includes high rates of physical restraints [14], pressure ulcers, antipsychotic medications without indication [15], falls, and frequent transfers to acute care settings [16]. LTC is a critical site of care for older adults and given the vulnerability of those individuals and the breadth of quality issues, it must be a high priority for research [17]. The aim of this study is to describe the priority setting methods we used with citizens and stakeholders to identify priorities for analysis within a well-established health services research program and more broadly, to gain insight into future LTC research directions.

## Methods

### Study design

To address our specific goals, we adapted the priority setting methods described by the James Lind Alliance (JLA) [18]. Typically, JLA priority setting partnerships bring together patients and clinicians to identify unanswered research questions in a given clinical area; the list of finalized priorities is then released publicly with the goal of influencing funding agencies and researchers [4, 19, 20]. Priority setting methods include a widely distributed survey with open ended questions on a specific clinical area to generate ideas for research, response management which includes collating and removing duplicates, literature searches to determine if sufficient research has been conducted on responses, and an in-person workshop with approximately 20–25 respondents to identify the final priorities.

### Context: translating research in elder care program

Translating Research in Elder Care (TREC), established in 2007, is a longitudinal pan-Canadian research program with a mission to improve the quality of care provided to LTC home residents and the quality of work-life for their paid caregivers [8, 21, 22]. A core value to TREC’s work is partnership with research end-users. The team includes approximately 40 researchers from across Canada, the United States, the United Kingdom, and Sweden, and 20 provincial and health region stakeholders. Researchers and stakeholders In 2016, TREC’s engagement strategy was expanded to include a citizen advisory committee comprised of LTC residents, potential future residents (specifically individuals living with a dementia), and family/friend caregivers to people living in LTC. The committee adopted the name Voices Of Individuals, family and friend Caregivers Educating uS (VOICES) includes members from across Canada and was established in recognition of the need for the voices of persons with lived experience to truly meet TREC’s commitment to integrated knowledge translation. TREC and VOICES jointly decided to refer to VOICES members as citizens, rather than patients. In dementia research, the term citizenship is used to reflect the personhood, visibility, voice, and inclusion of persons with lived experience [23–25]. Citizen in this context reflects the proactive engagement of persons with lived experience in efforts to enact social change [23]. This decision to adopt the term citizen also reflects the team’s desire to move away from the medical language of ‘patient’ which often refers to persons receiving care in acute care settings. VOICES was initially intended to play an advisory role [26, 27] but members expressed their desire to move to a more fully partnered position and were particularly interested in opportunities to provide advice on the development of new research projects. We now work with them as team members and their influence on our work, while sometimes difficult to quantify, can be felt at any gathering for which they are present. They, along with other TREC team members, were concerned that the rich database TREC had worked to collect was not being used to its full potential for secondary analyses. VOICES interest in seeing fuller use of the TREC data and their desire to become more involved in project generation led TREC to undertake an internal priority setting process to engage VOICES and other stakeholders to jointly identify 10 priority research questions that could be addressed using TREC’s existing longitudinal data repository.

### TREC data

The priority setting focused on questions that could be answered within the available TREC data. TREC data come primarily from 97 LTC homes in 5 health regions across 3 Western Canadian provinces (British Columbia, Alberta, Manitoba). LTC homes were randomly selected to be representative of those in urban areas and are proportionally stratified on bed size and ownership type (public-not-for-profit, private-for-profit, voluntary not for profit) [21, 28]. The TREC team administers a suite of survey instruments (known as the TREC Survey) to staff (regulated, unregulated, social workers, dieticians, pharmacists, rehabilitation therapists, recreation therapists/aids, managers) within all participating LTC homes. This Survey consists of validated measures of physical and mental health, burnout, empowerment, work environment, organizational citizenship behaviours, job satisfaction, and individual staff demographic characteristics. Within the survey is the Alberta Context Tool, a survey based instrument, developed and validated by TREC, which is used to assess staff’s work environment [29, 30]. After 5 waves of data collection, we have information from over 339 care units, 927 nurses, 4158 care aides, and 842 care staff (social workers, dieticians, pharmacists, rehabilitation therapists, recreation therapists/aids, managers). TREC also captures resident data from the participating LTC homes using the Resident Assessment Instrument – Minimum Data Set (RAI-MDS 2.0): The RAI-MDS 2.0 is a comprehensive clinical assessment instrument that has been mandated for completion on all LTC residents in nearly every Canadian province. The instrument includes over 400 items on measures such as cognition, physical function, behaviour, mood, and clinical signs and symptoms [31, 32]. As of April 2020, RAI-MDS 2.0 assessments from nearly 60,738 residents were held in the TREC data. All data are linkable and can be used to create comprehensive, longitudinal datasets that include information on residents, staff, care units, and LTC homes. The breadth of TREC data, the ability to identify care units and validly assess care units’ context or work environment, and its longitudinal nature make it a unique resource for on-going clinical trials in quality improvement and secondary analyses.

We used the JLA priority setting approach to identify research questions within the existing TREC data. We had to modify the JLA approach for these specific reasons: 1) participants were TREC stakeholder and partners, specifically decision makers (representatives from provincial ministry of health, regional health authorities), VOICES members, LTC home owner-operators, and other agencies engaged with TREC, rather than a broader public or clinical community; 2) our focus was specifically on identifying priority research questions that could be addressed with existing TREC data, and not an open discussion of general unanswered research questions regarding LTC homes; and 3) the final priorities are intended to be used by TREC investigators and trainees with TREC data.

### Participants: online survey

We administered an online survey to all VOICES members, TREC decision makers (regional health authority leaders, provincial health leaders), LTC owner-operators, and other relevant agencies associated with TREC. We did not include TREC researchers because the goal of the priority setting was to identify citizen and stakeholder research priorities, not researcher priorities. We instructed recipients that they could forward the survey to others in their network who were interested and involved in the TREC program, specifically. Survey recipients received a link to the survey and a reminder of our focus on research questions that could be addressed using TREC’s existing data (and not necessarily all research in LTC). The survey (see Supplementary Material) was divided into 5 sections, each focused on a different key aspect of the available TREC data (resident, staff, work environment, care unit, and facility). Within each section, we provided a short table outlining the main data elements. Questions were asked in the form of: “What questions do you have about LTC residents?” Participant responses were open-ended in free text boxes and respondents could list as many questions as they wanted (or leave blank). VOICES members were involved in the development and final review of the survey before it was distributed to respondents.

### Data collection: online survey

Our online survey was administered from October to December 2018. Data collection consisted of three email messages (one welcome, two reminder). The survey was hosted by SimpleSurvey™, a Canadian survey vendor (https://simplesurvey.com/). Once the survey closed, a steering committee, led by two investigators (AG, SC) and one research assistant worked together to categorize the survey responses. This researcher-led steering committee was critical since committee members needed to have substantial knowledge of the data in order to determine which questions were possible within the existing TREC database.

### Participants: final workshop

We held a face-to-face workshop in March 2019. The purpose of the workshop was for the attendees to come to consensus on the unranked top 10 priority research questions to be addressed with the TREC data. Ahead of the workshop, the attendees were sent a list of 34 research questions derived from the steering committee’s analysis of the survey responses. They were asked to rank the research questions in order of priority and to email us their ranked list and bring their ranked list with them on the day of the workshop. Twenty-one individuals were invited to the final workshop, two cancelled at the last minute.

### Data collection: final workshop

The workshop was facilitated by an experienced facilitator familiar with TREC but not involved in the research, and who was supported by two additional trained facilitators external to TREC. None of the TREC researchers or TREC trainees were in attendance, including the authors of this paper, to ensure that the researchers did not influence or bias the priority setting process. The workshop agenda was highly structured, and consensus was reached using the Nominal Group Technique, a format described by the JLA [33]. In the Nominal Group Technique, each group member states their opinion, without justification or explanation, and once all members have had a turn a moderated discussion follows. The goal is to allow each group member to have an opportunity to express their own views while minimizing opportunities for individual members to dominate the discussion. This is then followed by voting or ranking with structured group discussions [33].

In the morning, attendees were divided into three small groups. During this small group time, everyone shared his or her top and bottom three research questions, followed by discussion of their rationale. This ensured that each person was given dedicated time to talk and was intended to minimize power imbalances. The groups were then asked to rank order all 34 research questions. Over lunch, the rankings from each of the three small groups were aggregated to create a new ranked list. In the afternoon, attendees were assigned to new small groups. They were presented with the new ranked list of research questions and asked to re-rank, focusing on the top 15 research questions, as needed. Each group’s re-ranked list was then aggregated to create the penultimate ranked list of 34 research questions. The new list was presented to attendees who then had the opportunity for final large-group discussion. Any suggested changes were voted on with a majority decision.

At the end of the workshop, we distributed a workshop evaluation to all participants. The evaluation consisted of closed and open-ended questions related to their experience during the in-person workshop. Participants were asked to indicate their level of agreement on ten questions (1 = Strongly disagree, 2 = Disagree, 3 = Neither agree or disagree, 4 = Agree, 5 = Strongly agree).

### Analysis

The steering committee followed this process to analyze the online survey responses and prepare the final list of questions for the in-person workshop. First, since respondents could write multiple suggestions within a text box, unique questions were extracted. Second, the steering committee members removed responses that could not be assessed using the existing TREC data (considered “out-of-scope”) or if they coincided with existing TREC research. We retained all the out of scope suggestions for future projects. Third, the remaining suggestions were grouped into broad themes. This thematic assessment allowed us to identify duplicate or similar suggestions that could be merged. From these themes we developed an initial set of questions. Finally, two team members (AG, SC) refined the research questions in an iterative review process which assessed potential overlap in the questions and the ability to clearly assess the question using the available data. In this final step, we also further removed questions that duplicated on-going TREC research and/or were deemed out-of-scope on additional inspection. The JLA recommends a literature review to ensure that suggested research questions are unanswered. We did not conduct this review of the literature because the aim of the priority setting was to specifically address citizen and stakeholder questions using the existing TREC data, not to determine if there was enough research on the topic in the peer-reviewed literature. Instead, we focused on ensuring that research questions had not already been addressed using TREC data, rather than more broadly in the LTC home research literature. At the end of the process, we created a list of research questions derived from the suggestions provided by TREC’s partners and stakeholders in the online survey.

We analyzed the in-person workshop evaluations using descriptive summary statistics for the quantitative response items. The open-ended responses are presented in their entirety in the following results section.

## Results

We received 72 online survey responses (Table 1). The survey was distributed to 181 individuals, resulting in a 40% response rate. Respondents were primarily LTC home managers or administrators (34.7%) or family members or friends of a person living (or who had lived) in a LTC home (23.6%). We had 19 individuals attend the in-person workshop there were representatives from the following groups: 9 VOICES members, 1 direct care provider, 1 provincial ministry of health representative, 2 LTC owner-operators, 4 provincial health authority representatives, and representatives from 2 provincial associations (nursing, continuing care).

**Table 1:** Online survey respondent roles

Primary Role	N (%)
Regional or health authority policymaker	9 (12.5)
Provincial policymaker	7 (9.7)
LTC home manager or administrator	25 (34.7)
Family member or friend of LTC resident (past or present)	17 (23.6)
Person living with a dementia	1 (1.4)
LTC home staff	6 (8.3)
Other	7 (9.7)

**Fig. 1:**
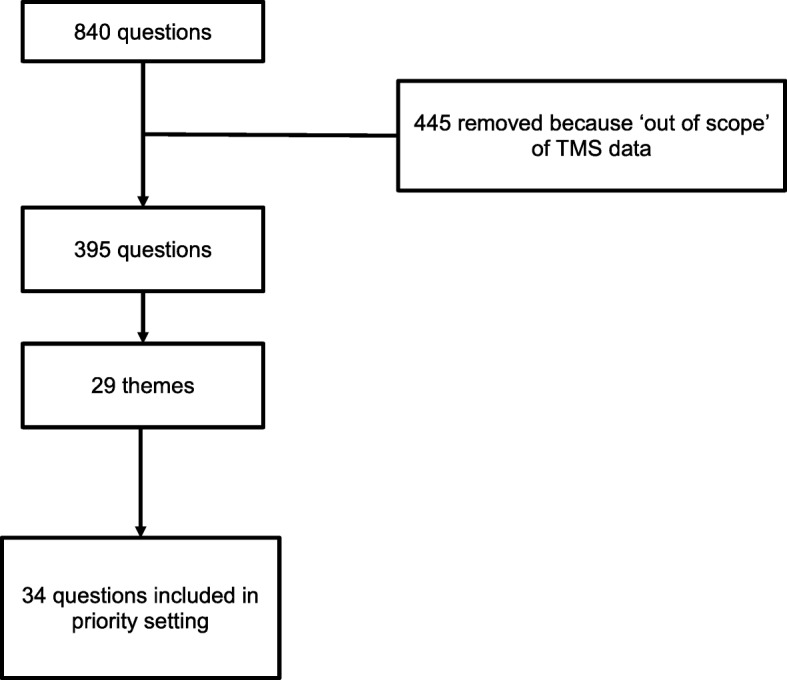
Research question development flow chart

The 72 online survey responses resulted in a total of 840 individual suggestions for research questions. We removed 445 responses that were out-of-scope (could not be addressed with existing TREC data) (Fig. 1). Topics that could not be answered with existing TREC data included: quality of life, resident expectations for care, engagement of family and friends, staff motivation and dedication, and questions related to resident or staff race and ethnicity. These out-of-scope questions, while not included in the final list of priorities, will help inform future data collection and new project development. The steering committee refined the remaining 395 suggestions to identify overlap with existing projects and other aspects of feasibility given TREC’s data. The final list of 34 research questions was created from the 395 suggestions (see Supplementary Material for list of all 34 questions).

At the end of the day, a top 10 list of agreed upon research priorities was generated. Two suggested changes were made but only one was approved by the group. The unranked final 10 questions (Table 2) focused on relationships between staffing (e.g., numbers, mix, type) and resident health outcomes, quality improvement activities and resident and staff outcomes, and resident quality indicators (e.g., pain, physical restraint).

**Table 2:** Ten research priorities for TREC

1	What is the relationship between staffing levels and staff mix with resident outcomes (e.g. responsive behaviours)?
2	Is there an association between engagement in quality improvement activities and resident outcomes (e.g. responsive behaviours, falls)?
3	Is there an association between access to medical care (e.g. physicians and nurse practitioners) and resident outcomes?
4	Are care aides included in decision making about residents?
5	Is there an association between the work environment (including physical space) and resident outcomes (e.g. responsive behaviours)?
6	Is there an association between engagement in quality improvement activities and staff’s quality of work life and work environment?
7	What resident characteristics are associated with physical restraint use?
8	What resident characteristics are associated with quality of work life (e.g. burnout) and work engagement?
9	What leader (e.g. LPN vs RN leader) and leadership qualities are associated with a positive work environment?
10	Is there an association between resident pain and other quality indicators?

We evaluated participants’ experience during the final workshop, 18 out of 19 participants provided feedback (Table 3). Participants felt comfortable in the small group (Mean = 4.9) and large group (Mean = 4.9) discussions. They felt that their voice was heard throughout the day (Mean = 4.8). Citizen and stakeholder participants had positive feedback about the priority setting process, particularly on the organization of the final workshop. Although attendees came from a variety of professional and personal backgrounds, participants felt that the structure of the in-person meeting (small groups with dedicated time for each person to speak) ensured that their opinions were listened to and valued. Table 4 includes all the open-ended responses to the workshop evaluation.

**Table 3:** Priority setting workshop evaluation results

	Mean Response^a^
I was clear on the purpose of today’s Priority Setting Workshop	4.7
The ranking sheet (that you received via email) was clear and understandable)	4.4
The presentation of how the questions were developed was valuable	4.5
There was sufficient time allotted for the small group ranking	4.3
The discussion in the small group was valuable	4.7
I felt comfortable in the small group discussions	4.9
I felt comfortable in the large group discussion	4.9
The length of the workshop (i.e., 10 am to 3 pm) was appropriate	4.5
I felt my voice was heard through the discussions	4.8
I feel that this was a valuable use of my time	4.7

^a^Possible scores ranging from 1 to 5, with 5 meaning ‘strongly agree’

**Table 4:** Workshop evaluation open-ended responses

**Strengths of the priority setting process**	
Enough VOICES “voices” - great (Not just one group)	
Collaboration process valuable to help us come to consensus	
Great to have the mixture of attendees. Really appreciate the focus of the event; good to have an “outside” facilitator	
The pre-meeting with VOICES was a great primer for the session.	
The facilitator did an excellent job a explaining the priority setting process. The small groups were excellent and I feel like everyone was able to share valid and important information	
Good timing, led to sharing of many thoughts, ideas	
Very well facilitated and organized. Appreciated the varied perspectives and feedback in determining the priorities	
Great sharing of thoughts and different perspectives	
It was tough to rank 34 items, it might have been clearer with fewer items	
The inclusive nature of the session was very valuable	
Great work in getting to a final product. Outside facilitation was especially good since she focused on the task and no vested interest	
It was very informative to have the various viewpoints represented at the table	
**Suggestions for improving the priority setting process**	
Would have liked to see more direct care people (care managers/admin/RNs) attending the priority setting exercise	
When providing a ranking sheet again provide a bit more explanation. Process familiar perhaps to creator, not so much so for rater	
I found 2nd grouping instructions lacked clarity … felt longer harder. We felt success with Group 1 then undone for Group 2. Hard to let go.	
Please incorporate this methodology more often in TREC, as applicable in determining priorities or where decisions need to be made	
Too many options /wording similarities	
Sharing of data between TREC and provincial/health authority team	
Have 2 days where 1 day rank half then have time that night to think about what to say for top 10	
Include some staff is possible (HCAs, LPNs, RNs) as their input would present perspectives that are very valuable	

## Discussion

Our priority setting approach identified 10 areas for secondary analyses in the TREC program and future research more broadly in the LTC sector. General themes that emerged from our list of questions focused on the staff (e.g., ratios, type of care provider), work environment on resident outcomes and the impact of quality improvement activities on residents and staff. Our findings are unique because they offer specific insights into the type of questions citizens and stakeholders are interested in from LTC research. Our list suggests that citizens and stakeholders are interested in non-clinical aspects of care in LTC homes. Additionally, our work shows that research teams can adapt the JLA process to help establish their research agendas in such a way that non-researcher team members can have a strong say in its direction and feel that their voices are heard in establishing these new directions.

We were unable to find comparable published priority setting exercises in the LTC sector or in established research teams. However, the Alzheimer Society of Canada recently conducted a priority setting partnership, asking individuals from across Canada to submit questions related to dementia [4]. This priority setting identified areas related to stigma, quality of life, health system capacity, and dementia care. These priorities offer broad areas for future research but did not align with our priorities which are more specifically targeted to the LTC sector (as opposed to the community) and the TREC program. Another recent priority setting in Canada was conducted by the Alberta Health Services Seniors Health Strategic Clinical Network™ in partnership with patients, caregivers, and clinicians in Alberta to prioritize questions about older adults’ health and healthcare [34]. Their top ten included strategies for older adults to remain independent, how the healthcare system can encourage prevention of disease and disability, improving rural older adults’ access to care, and increasing availability of dementia-related care and service. Taken together, the national and provincial priority setting results offer important direction for research and funding priorities but are broad, not connected to any existing research team activities, and are not specific to any specific setting of care. Identifying priorities within an existing program of research allowed participants to pose more specific questions that could be reasonably answered without new data collection. Furthermore, our priority setting approach included owner-operators and health system decision makers which brings a different level of perspective and need for information from typical priority settings, including those mentioned in the Introduction and above, which have been largely focused on a single disease.

We adapted the JLA method to enable us to identify research questions within an established data repository. This approach, prioritizing research in an existing research program, offered an opportunity for our citizens and stakeholders to direct the research agenda. To our knowledge, this is the first type of study that modified the JLA method in this way. The JLA methods do not typically restrict participants to specific research foci and therefore there were no best practices for the survey development or analysis. We found that restricting participants to questions answerable within the TREC data posed some challenges. Although we provided details of the TREC data holdings, reading through the list of variables and topics in the existing data may be overwhelming. Nearly 50% of the suggestions provided by respondents during the initial survey were classified as out-of-scope and could not be answered by the existing TREC data. Rather than considering these questions unanswerable in our existing data, these out-of-scope questions point to specific areas for future research efforts that were generated entirely by citizens and stakeholders. Another modification we made, was to omit the literature reviews as recommended in the JLA. For our purposes, we were not as interested in whether specific research questions had ever been addressed in the broader research literature but rather had they been already addressed using the TREC data, which would lead to findings more relevant to TREC’s citizen and stakeholder partners. While we did not conduct systematic reviews to assess whether each of the identified research priorities have been addressed before, TREC researchers with extensive expertise in the content area of prioritized research questions have identified that the identified priorities indeed not only constitute a gap in using our TREC data, but also a gap in the literature. For example, the question identified as highest priority (influence of different staffing levels and staff mix on resident outcomes) has been addressed in LTC settings [35, 36], but these reviews highlight that the quality of available studies is weak, findings are heterogeneous and inconclusive, and that especially the interaction between elements of care staff work environments (leadership, culture, connections within the team) and staffing levels or staff mix may better explain resident outcomes. However, this interaction has not been studied yet in LTC. Our TREC data is suited to address this gap and team of TREC researchers has started to do that research. This modified priority setting process may be a useful method for research teams that want to take use their existing data and to engage non-researchers in the process of project and research question development.

We found that the design and conduct of the priority setting workshop provided a unique opportunity for our non-research team members to identify their research interests. Participants noted that preconceived hierarchies related to the addition of stakeholders from various provincial ministries or health authorities were limited due to the nominal ranking process and the expert facilitation of the working groups. Engaging experienced facilitators was critical to the success of the in-person workshop. Prior to the in-person meeting, our team circulated an agenda and a list of attendees, including name, position, and organizational affiliation. VOICES members indicated that knowing the attendee list ahead of time was helpful to understanding who was in the room and familiarize themselves with the different types of professional expertise that attended the workshop. We did receive some negative feedback from participants regarding the in-person workshop. In the evaluation, some participants described that they felt frustrated when their small working group lists were re-ordered following the amalgamation of all the small group responses, especially given how hard the groups had worked to reach consensus. Were we to do this priority setting again, we would have prepared participants for the potential for this emotional reaction when their initial lists undergo re-ordering during the large group discussion.

### Limitations

This study has limitations. We had only a 40% survey response rate. This lower response rate may have been due to the structure of the survey. In order to describe the data holdings, we included details about the survey instruments. Although we aimed to limit the survey details, this may have increased the cognitive burden and resulted in a lower response rate. We would have liked to have more stakeholders involved (e.g., directors of care, care aides, non-VOICES citizen representatives) in the final workshop, however we were unable to expand the scope of stakeholder involvement at workshop given limits to overall number of attendees based on JLA guidelines and the need to include all existing team members (i.e.,11 VOICES members). We did not have any citizen representatives that live in a LTC home. We continue to work with our stakeholder partners to identify new citizen team members who live in LTC homes; however, given the high level of need (including advanced cognitive impairment) and the difficult logistics of meaningfully including someone living in this setting, it was not possible at the time we undertook this project.

## Conclusion

Overall, this modified priority setting technique offered a useful way to hear from citizens and stakeholders about their research priorities, without the external pressures or involvement of researchers and trainees. As members of our team begin to develop projects with the aim of answering these research questions, we believe that using this method to generate research questions is a worthwhile approach to generate questions for secondary analysis and research question development for research teams.

## Supplementary information

**Additional file 1 Supplementary File 1.** Online survey content.

## Data Availability

The data generated during this study are available from the corresponding author on reasonable request.

## Abbreviations

LTC: Long-term care
TREC: Translating Research in Elder Care
VOICES: Voices Of Individuals, family and friend Caregivers Educating uS
