# Supplementary material for: Citizen and stakeholder led priority setting for long-term care research: identifying research priorities within the Translating Research in Elder Care (TREC) Program
Source: Res Involv Engagem. 2020 May 15;6:24. doi: 10.1186/s40900-020-00199-1 (PMC7229578; doi:10.1186/s40900-020-00199-1)
Supplement: Supplementary file 1 — Additional file 1 Supplementary File 1. Online survey content. [file 40900_2020_199_MOESM1_ESM.docx]

**Supplementary File 1: Online survey content**

Welcome to the TREC Priority Setting Survey. The purpose of this survey is to hear your thoughts on the kinds of research that you would like to see done using the TREC Measurement System – also known as TREC Database. The TREC Database is all of the data that TREC has collected since its start in 2007.

This survey is divided into 6 sections. The first 5 sections are divided into the main types of data that TREC collects: 1) resident, 2) staff, 3) work environment, 4) care unit, and 5) facility. Within each of these sections, you will find more specific information about what data are collected by TREC. You will then be prompted to write what kinds of questions or ideas you have for each section. In the final section of the survey, you will be asked to add your final thoughts or areas of interest, even those that we may not be able to research using the TMS. The thoughts, ideas, and questions that you write in this survey will help us to better understand what kinds of research questions are of greatest priority to TREC’s partners.

These priorities will then be presented to TREC researchers and trainees for future research projects.

Finally, we want you to know that there are no right or wrong, or good or bad answers. We want to know what you think is important based on your experiences and perspectives, and what you know about TREC. If you do not have anything to add to a certain section, it is okay to leave questions blank.

Thank you for taking the time to complete this survey.

**Section 1: Residents**

Resident data were collected using the Resident Assessment Instrument – also known as the RAI-MDS 2.0. The RAI-MDS 2.0 is a clinical assessment that nursing home care teams complete on each resident. Care teams (often led by registered nurses) complete the RAI-MDS 2.0 using information from residents’ charts and other sources (for example, talking to other staff). RAI-MDS 2.0 assessments are completed at least once every 3 months on each resident. The RAI-MDS 2.0 includes information on the following areas:

| **Type of Information** | **Additional Details** |
| --- | --- |
| Demographic information | Sex, age, date of admission to nursing home |
| Diagnoses | A checklist of 60 medical diagnoses such as dementia, diabetes, heart failure, and past stroke |
| Physical symptoms | Includes over 15 symptoms such as pain, shortness of breath, and vomiting |
| Mood | Includes both diagnosis of depression (in the diagnostic checklist) and individual depressive symptoms |
| Degree of cognitive impairment | Measured in a way to distinguish between none, mild, moderate, and severe impairment |
| Degree of physical impairment | Measured by the ability to perform “Activities of Daily Living” (such as feed self and get dressed) |
| Communication difficulties | Hearing, vision, speech: Includes difficulties understanding others and difficulties expressing self |
| Presence of responsive behaviours | Includes physically and verbally abusive behaviours, socially inappropriate/disruptive behaviours and resisting care |
| Certain treatments | Includes treatments related to nutrition (such as feeding tube use), skin care, special services (such as physical therapy), and certain medication classes (such as antipsychotic medications) |
| Recent falls | Asks about falls in the prior 30 and the prior 180 days |

The RAI-MDS 2.0 does **NOT** include any information on the following areas:

- Ethnicity, race, or religion
- Quality of life
- Level of engagement of family or friend caregivers
- Preferences for care

Questions:

What would you like to know about the people who live in nursing homes? This can include questions about how the population has changed or how residents’ needs change over time. Please list any/all ideas, thoughts, or questions below.

**Section 2: Staff Information**

During the TREC Survey, the TREC data collectors ask nursing home staff a number of questions about themselves and their experiences at work. Nursing home staff include care aides, nurses, allied health providers, managers and administrators. Data from physicians were collected only during Waves 1 and 2 of the TREC Survey due to very low responses. The TREC Survey includes information from staff on the following areas:

| **Type of Information** | **Additional Details** |
| --- | --- |
| Demographic information | Age, sex, first language |
| Education | Certificate, Degree, Country of Graduation |
| Number of years of work experience | Overall and in that job at time of survey |
| Number of jobs | Number of jobs working at time of survey |
| Attitudes toward work and research use | Opinion expressed (positive to negative) towards research knowledge |
| Job satisfaction | Feelings about job |
| Commitment to work | Dedication and interest in job |
| Organizational citizenship | Individual efforts to improve the work place |
| Exposure to responsive behaviours | Includes threats, physical assault, and unwanted sexual attention |
| Burnout-negative (emotional exhaustion and cynicism) | Unrelieved work stress that includes feeling strained, tired, less enthusiastic about work |
| Burnout-positive (professional efficacy) | Feeling good at the job, feeling accomplished, feeling that their accomplishments in the job are worthwhile |
| Self-reported health | Physical health and mental health as reported by the individual |
| Tasks left undone | Tasks that were not completed during most recent shift due to lack of time |
| Tasks that were rushed | Tasks that were rushed through during most recent shift due to lack of time |

The TREC Survey does **NOT** include any data on the following:

- Ethnicity, race, or religion

Questions:

What would you like to know about the people who work in nursing homes? This can include questions about care aides, nurses, or other kinds of staff, as well as questions about how they have changed over time. Please list your ideas/thoughts/questions below.

What would you like to know about how the characteristics of nursing home staff affect residents? Please list your ideas/thoughts/questions below.

What would you like to know about how the characteristics of residents affect the nursing home staff? Please list your ideas/thoughts/questions below.

**Section 3: Work Environment**

As part of the larger TREC Survey, nursing home staff answer questions that help us to understand their work environment. These data are collected using a special tool called the Alberta Context Tool, or ACT. In most cases, when staff answer questions about their work environment, they are talking about the care unit on which they work. The ACT asks questions about the following areas that make up the work environment:

| **Type of Information** | **Additional Details** |
| --- | --- |
| Leadership | Describes the actions of leaders on the unit to whom the care aides report most often (such as asks for feedback, actively listens, mentors, resolves conflicts) |
| Culture | Describes the experience working on the unit (such as whether staff feel they have control over their work, feel supported, and receive recognition for their work) |
| Evaluation (Feedback) | Describes the process of receiving feedback data on performance and creating a plan of action |
| Formal interactions | Describes scheduled exchanges of information such as meetings, reports, resident care conferences, family conferences |
| Informal interactions | Describes unscheduled/unplanned exchanges of information such as hallway conversations or huddles |
| Social capital | How connected people feel to their co-worker teams |
| Structural resources | Access to resources (e.g., library, text books, journals, notice boards, policies and procedure manuals) in the work area to do the job or work tasks |
| Organizational slack | Perceptions of available resources or time to adapt workflow, as needed  Measured on 3 areas: staffing, space, and time |

Questions:

What would you like to know about the work environment in nursing homes? This can include questions about how the work environment affects staff and questions about how the work environment affects residents. Please list your ideas/thoughts/questions below.

**Section 4: Care Unit Information**

As part of the TREC Survey, nursing home managers answer questions specifically about the care units. These questions largely focus on how care units are staffed and structured. Available information on care units includes the following areas:

| **Type of Information** | **Additional Details** |
| --- | --- |
| How often physicians are on the unit | Number of physicians who visit the unit and if they make routine visits |
| How often nurse practitioners are on the unit | Number of nurse practitioners who visit the unit and if they make routine visits |
| Number of staff scheduled on the unit (on days, evenings, nights) | Average number of staff scheduled for each Monday-Friday, Saturday, and Sunday |
| Students doing work placements on unit | Nurse, other staff, care aides completing placements |
| Major near misses | This refers to an event that could have caused death or significant harm but was caught before anything happened |
| Work process improvements | Changes made by the unit leader to improve how people on the unit work |
| Care Aide Hours Worked | Number of care aide hours worked per resident day |
| Licensed Practical Nurse (LPN) Hours Worked | Number of LPN hours worked per resident day |
| Registered Nurse (RN) Hours Worked | Number of RN hours worked per resident day |

Questions:

What would you like to know about care units in nursing homes? This can include questions about how units are staffed or structured and questions about how this relates to the work environment. Please list your ideas/thoughts/questions below.

What would you like to know about the relationship between care units and characteristics of staff? This can include questions about staff training or feelings about work. Please list your ideas/thoughts/questions below.

What would you like to know about the relationship between care units and characteristics of residents? This can include questions about resident health conditions or outcomes. Please list your ideas/thoughts/questions below.

**Section 5: Nursing Home Facility Information**

As part of the TREC Survey, nursing home administrators complete questions about the nursing home facility itself. These questions largely focus on structural aspects of the nursing home. Information on nursing homes is shown in the table:

| **Type of Information** | **Additional Details** |
| --- | --- |
| Year facility was built |  |
| Owner-operator type | Private for profit, public not for profit, and voluntary (non profit, often faith based) |
| Available services and programs | Examples include geriatric mental health consulting, pharmacy, dental care, foot care, and pastoral care |
| Quality improvement activities   - Regular use of data for improvement activities - Meetings with unit leadership on quality improvement activities - Meetings external to the nursing home on quality improvement activities - Education sessions on quality improvement activities | Questions ask about the occurrence of each of these things: daily, weekly, monthly, quarterly, or annually. |

Questions:

What would you like to know about nursing home facilities? This can include questions about the relationship between the nursing home facility and the work environment, characteristics of the staff who work there, or the residents who live there. Please list your ideas/thoughts/questions below.

**Section 6: Additional questions**

There are of course many things not in the TREC database at this time. Is there anything else that you would like to know about nursing homes, their staff, or their residents that likely cannot be addressed with existing TREC data? This can include questions that you know we cannot answer using the existing TREC data or are unsure if we can answer using the data. Please list your ideas/thoughts/questions below.

How do you identify yourself? Check all that apply.

- Family member or friend to someone living in a nursing home (either now or in the past)
- Person living with dementia
- Nursing home staff
- Nursing home manager or administrator
- Regional or health authority policymaker
- Provincial policymaker
- Other (specify: )

**Supplementary File 2: 34 research questions (prior to in-person workshop)**

| Research Question |
| --- |
| What is the relationship between different staffing levels and staff mix on resident outcomes (e.g. responsive behaviours)? |
| Is there an association between engagement in quality improvement activities and resident outcomes (e.g. responsive behaviours, falls)? |
| Is there an association between access to medical care (e.g. physicians and nurse practitioners) and resident outcomes? |
| Are care aides included in decision making about residents? |
| Is there an association between the work environment (including physical space) and resident outcomes (e.g. responsive behaviours)? |
| Is there an association between engagement in quality improvement activities and staff’s quality of work life and work environment? |
| What resident characteristics are associated with physical restraint use? |
| What resident characteristics are associated with quality of work life (e.g. burnout) and work engagement? |
| What leader (e.g. LPN vs RN leader) and leadership qualities are associated with a positive work environment? |
| Is there an association between resident pain and other quality indicators? |
| Is there an association between presence of allied staff (e.g. occupational therapists) and resident outcomes? |
| Is there an association between staffing levels/mix and tasks rushed/left undone? |
| Has the profile of residents changed over time (on admission and overall)? |
| Is there an association between staff work engagement and resident quality indicators? |
| What are current staffing levels and patterns including use of agency staff, weekends, nights, etc.) and how have these changed over time? |
| How do residents who are younger than 65 differ from those who are 65 or older? |
| What is the relationship between direct care hours and resident outcomes? |
| How do different staff types (e.g. care aides, LPNs, registered nurses, managers, specialists) differ in their characteristics and measures of quality of work life? |
| Is there an association between resident characteristics and tasks rushed/left undone? |
| What is the relationship between direct care hours and quality of staff work life? |
| Is there an association between staff burnout and resident outcomes? |
| How do specialized care units differ from general units |
| Is there an association between staff physical health and resident outcomes? |
| Have there been changes in staff work engagement over time? |
| What are the factors that are associated with staff communication? |
| What type of education and resources are available to staff? |
| What is the frequency of adverse events/near misses and does this vary across long-term care homes? |
| What is the relationship between different staffing levels and staff mix on staff quality of work life (e.g. burnout)? |
| Do staffing levels and staffing patterns differ by facility ownership type? |
| Is there an association between resident case-mix and staff absenteeism? |
| Does facility ownership type impact the work environment? |
| What are the predictors of staff’s length of service? |
| Have there been changes in antipsychotic use (and associated adverse events such as falls) following the implementation of the Alberta antipsychotic initiative? |
| Is facility ownership type associated with resident outcomes (quality indicators, responsive behaviours, falls)? |
